# Effect of β-Hydroxybutyrate on Autophagy Dynamics During Severe Hypoglycemia and the Hypoglycemic Coma

**DOI:** 10.3389/fncel.2020.547215

**Published:** 2020-09-23

**Authors:** Carmen Torres-Esquivel, Teresa Montiel, Marco Flores-Méndez, Lourdes Massieu

**Affiliations:** Departamento de Neuropatología Molecular, División de Neurociencias, Instituto de Fisiología Celular, Universidad Nacional Autónoma de México, Ciudad de México, Mexico

**Keywords:** hypoglycemia, ketone bodies, neuronal death, autophagy, AMPK

## Abstract

Glucose supply from blood is mandatory for brain functioning and its interruption during acute hypoglycemia or cerebral ischemia leads to brain injury. Alternative substrates to glucose such as the ketone bodies (KB), acetoacetate (AcAc), and β-hydroxybutyrate (BHB), can be used as energy fuels in the brain during hypoglycemia and prevent neuronal death, but the mechanisms involved are still not well understood. During glucose deprivation adaptive cell responses can be activated such as autophagy, a lysosomal-dependent degradation process, to support cell survival. However, impaired or excessive autophagy can lead to cell dysfunction. We have previously shown that impaired autophagy contributes to neuronal death induced by glucose deprivation in cortical neurons and that D isomer of BHB (D-BHB) reestablishes the autophagic flux increasing viability. Here, we aimed to investigate autophagy dynamics in the brain of rats subjected to severe hypoglycemia (SH) without glucose infusion (GI), severe hypoglycemia followed by GI (SH + GI), and a brief period of hypoglycemic coma followed by GI (Coma). The effect of D-BHB administration after the coma was also tested (Coma + BHB). The transformation of LC3-I to LC3-II and the abundance of autophagy proteins, Beclin 1 (BECN1), ATG7, and ATG12–ATG5 conjugate, were analyzed as an index of autophagosome formation, and the levels of sequestrosome1/p62 (SQSTM1/p62) were determined as a hallmark of autophagic degradation. Data suggest that autophagosomes accumulate in the cortex and the hippocampus of rats after SH, likely due to impaired autophagic degradation. In the cortex, autophagosome accumulation persisted at 6 h after GI in animals exposed to SH but recovered basal levels at 24 h, while in the hippocampus no significant effect was observed. In animals subjected to coma, autophagosome accumulation was observed at 24 h after GI in both regions. D-BHB treatment reduced LC3-II and SQSTM1/p62 content and reduced ULK1 phosphorylation by AMPK, suggesting it stimulates the autophagic flux and decreases AMPK activity reducing autophagy initiation. D-BHB also reduced the number of degenerating cells. Together, data suggest different autophagy dynamics after GI in rats subjected to SH or the hypoglycemic coma and support that D-BHB treatment can modulate autophagy dynamics favoring the autophagic flux.

## Introduction

The brain is a highly dynamic and energy-demanding organ that depends on the continuous glucose supply from blood, thereby disturbed glucose metabolism can lead to brain dysfunction and even brain injury (Mergenthaler et al., 2013). Reduced cerebral glucose delivery occurs during hypoglycemia, a condition considered as a major complication of insulin treatment in type 1 diabetes mellitus (DMT1) patients (Cryer, 2005). Patients can suffer two events of moderate hypoglycemia (60–40 mg/dl blood glucose) per week and one of severe hypoglycemia (SH, >35 mg/dl) per year. The presence of repeated episodes of moderate hypoglycemia increases the risk for SH, which can culminate in the state of coma resulting in irreversible brain damage in vulnerable brain regions, such as the cortex and hippocampus (Auer et al., 1984). Under conditions of limited glucose availability, such as ischemia, hypoxia, hypoglycemia, and cerebral trauma, alternative energy substrates to glucose, such as the ketone bodies (KB), acetoacetate (AcAc), and β-hydroxybutyrate (BHB), can be used by the brain (Melø et al., 2006) and prevent brain injury (Suzuki et al., 2002; Masuda et al., 2005; Puchowicz et al., 2008; Haces et al., 2008; Julio-Amilpas et al., 2015). KB are metabolized through the tricarboxylic acid cycle (TCA) and their protective effect has been attributed to enhanced cellular energy metabolism, improved mitochondrial activity, and decreased production of mitochondrial reactive oxygen species (ROS; Maalouf et al., 2007; Marosi et al., 2016). Other actions have been described for KB including epigenetic, antioxidant, anti-inflammatory, and the up-regulation of brain-derived neurotrophic factor; they activate ATP-sensitive potassium channels and have been described as signaling molecules (Newman and Verdin, 2014; Camberos-Luna and Massieu, 2020). In addition, we have recently reported that the D isomer of BHB (D-BHB) stimulates the autophagic flux during glucose deprivation and prevents neuronal death in cortical cultures (Camberos-Luna et al., 2016).

Macro-autophagy (here referred to as autophagy) can be activated during nutrient deprivation and is characterized by the formation of a double-membrane structure named autophagosome that subsequently fuses with a lysosome leading to the degradation of damaged proteins and organelles for the restoration of cell homeostasis (Klionsky and Emr, 2000; Yin et al., 2016). During nutrient deprivation, mTOR inhibition by AMPK leads to ULK1 S317 phosphorylation, which in turn phosphorylates Beclin-1 (BECN1) promoting the activity of class III PtdIns3K complex, initiating the formation of the nucleation site. On the contrary, during nutrient abundance mTOR activity phosphorylates ULK1 S757, preventing its activation by AMPK and autophagy initiation. Also, AMPK can directly phosphorylate ULK1 at S317 and initiate autophagy (Egan et al., 2011; Kim et al., 2011). The autophagy-related proteins (ATG) are key players in autophagosome formation and membrane expansion. Microtubule-associated light chain-3 (LC3) is cleaved by ATG4 and conjugated with phosphatidylethanolamine (PE) to produce LC3-II. LC3-II is associated with autophagosomes where it functions as a docking site of adaptor proteins such as SQSTM1/p62, which delivers polyubiquitinated proteins to the autophagosome for degradation. SQSTM1/p62 is degraded together with cargo by lysosome hydrolytic enzymes. ATG7 and ATG3 form the conjugation system involved in the relocation of LC3-I to the phagophore membrane and ATG5 covalently binds to ATG12 forming the ATG12–ATG5-ATG16 conjugate essential for autophagosome membrane elongation. Mature autophagosomes fuse with a lysosome leading to cargo degradation (Sou et al., 2008; Wirawan et al., 2012). Enhanced conversion of LC3-I to LC3-II and a reduction in SQSTM1/p62 abundance is taken as an index of autophagic flux.

Deficient autophagy in the central nervous system leads to the accumulation of ubiquitinated proteins, axonal degeneration, and neuronal death. Loss of cortical and cerebellar neurons has been observed in ATG7-deficient animals (Komatsu et al., 2005, 2006) and animals lacking ATG5 do not survive (Kuma et al., 2004). Also, excessive autophagy can lead to cell death, and its inhibition can prevent acute ischemic brain injury (Carloni et al., 2008; Fu et al., 2016; Wang et al., 2018). Autophagy dynamics during severe hypoglycemia *in vivo* has not been investigated, neither its role in selective neuronal death. Hence, we have studied the changes in autophagy markers during severe hypoglycemia (SH) without glucose infusion (GI), and during GI after SH or a brief period of coma. We also investigated whether D-BHB treatment in animals subjected to coma is associated with the preservation of functional autophagy, as we have previously observed *in vitro* (Camberos-Luna et al., 2016). We have evaluated the changes in the abundance of proteins involved in autophagosome formation and maturation, Beclin1 (BECN1), p-ULK1, ATG7, ATG12–ATG5, and LC3-II, and in SQSTM1/p62 as a marker of autophagy degradation. The role of AMPK and mTOR in autophagy activation was also investigated. Results suggest that in the cerebral cortex SH induced the accumulation of autophagosomes, which persisted 6 h after GI likely due to deficient autophagy degradation, and recovered at 24 h. In contrast, in the hippocampus, autophagosomes accumulated after SH but no change was observed after GI. In animals exposed to the hypoglycemic coma, significant autophagosome accumulation was observed at 24 h after GI in both brain regions. Besides, AMPK-dependent phosphorylation of ULK1 S317 was observed at this time in animals subjected to coma, suggesting autophagy activation. When D-BHB was administered after the coma, SQSTM1/p62 degradation was enhanced and ULK1 S317 phosphorylation was reduced, increasing cell survival. Data suggest that D-BHB attenuates autophagy activation and restores the autophagic flux promoting cell survival.

## Materials and Methods

Three-month-old male Wistar rats (280–320 g) were used throughout the study. They were obtained from the Instituto de Fisiología Celular (IFC) animal house, at the Universidad Nacional Autónoma de México (UNAM). The guide for the Care and Use of Laboratory Animals (NIH publications No. 80-23 Revised 1996) of the National Institute of Health was followed and animals were handled accordingly and with the approval of the Animal Care Committee (CICUAL) of the IFC (protocol number LMT160-20). The number of animals used was optimized and all efforts were made to minimize their suffering. Experiments are reported according to the ARRIVE guidelines (Animal Research: Reporting *in vivo* Experiments). Animals were kept with food and water *ad libitum* and under standard dark/light cycle and temperature conditions and housed in individual cages. A sample size of three to seven animals per group was used. Animals were randomly distributed among the different groups and at least one animal from each experimental group was included per experiment. Seventy-three animals were used for western blot, 28 for histology and immunocytochemistry determinations, and 21 for BHB blood determinations, as indicated in figure legends.

### Induction of Severe Hypoglycemia Without Glucose Infusion (SH)

**Figure 1:**
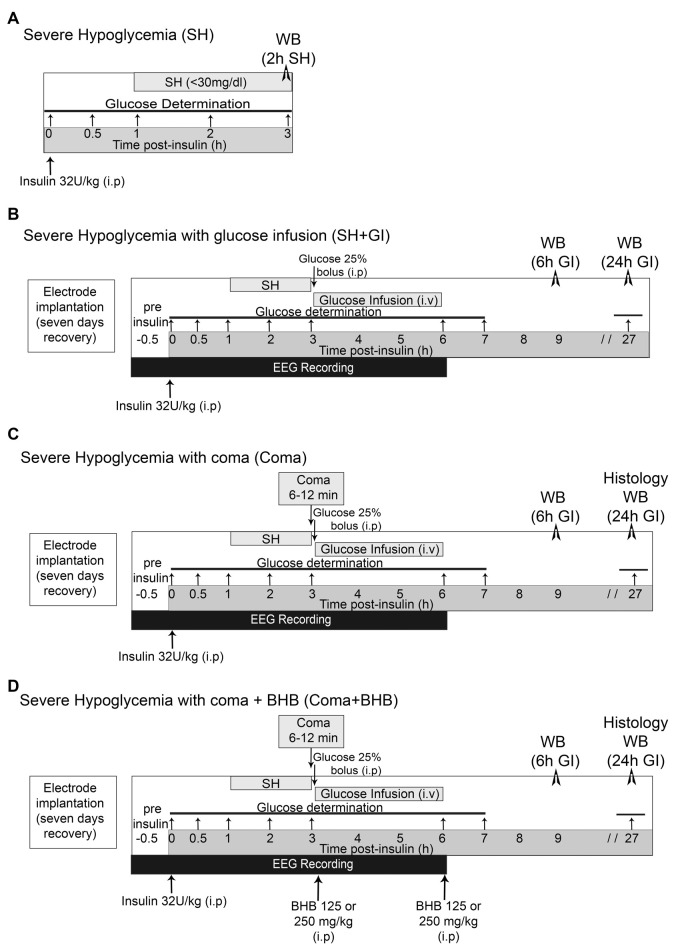
Induction severe hypoglycemia and the hypoglycemic coma. **(A)** Severe hypoglycemia (SH) group. SH was induced by the intraperitoneal (i.p.) injection of insulin (32 U/kg) and animals were euthanized 2 h after they reached 30 mg/dl blood glucose. **(B)** SH + GI group. Animals were implanted with electrodes 1 week before the induction of hypoglycemia for electroencephalogram (EEG) recording and were subjected to SH with glucose infusion (GI). Insulin was injected (i.p) and 2 h after animals reached SH, they were rescued with glucose before they fell into the coma state. **(C)** Coma group. Animals were treated identically as those from the SH + GI, but SH was left to progress to the coma state for 6–12 min and immediately after they were rescued with glucose. **(D)** Coma + BHB group. Animals were treated identically as the Coma group, but they received two doses of either 125 or 250 mg/kg D isomer of β-hydroxybutyrate (D-BHB; i.p.), the first 10 min after the onset of glucose infusion after the coma and the second at the end of glucose infusion glucose infusion. Animals were euthanized 6 or 24 h after GI in groups **(B–D)**.

Severe hypoglycemia (SH) was induced in the home cage in non-anesthetized animals partially fasted overnight (food restricted to four pellets) and during the experimental period. They received a single intraperitoneal injection of 32 U/kg human insulin (Lilly, Humulin 70/30, Indianapolis, IN, USA). Blood glucose was monitored from a blood sample obtained from the tail vein before (basal levels) and at 0.5, 1, 2, and 3 h after insulin injection using a standard glucometer (Abbott Laboratories, Bedford, MA, USA). One-hour after insulin administration, animals reached SH (<40–30 mg/dl) and were euthanized 2 h later (Figure 1A). Brains were extracted and prepared for western blotting.

### Induction of Severe Hypoglycemia With Glucose Infusion (SH + GI)

Animals were subjected to SH and rescued with GI. They were partially fasted overnight and during the whole experimental period. To monitor electrical brain activity through electroencephalogram (EEG) recording, 1 week before hypoglycemia animals were implanted with epidural electrodes under 2.0 to 3.0% isoflurane anesthesia. Meloxicam (1 mg/kg i.p.) was administered post-surgery as an anti-inflammatory. Animals were intraperitoneally (i.p.) injected (between 9:00 and 11:00 am) with 32 U/kg insulin to induce hypoglycemia. Blood glucose was monitored before (time 0) and at 0.5, 1, 2, 3, 6, 7, and 27 h after insulin. To determine basal brain electrical activity, EEG recording started 30 min before insulin administration and continued during the whole hypoglycemic and GI periods until the normal electrical activity was completely recovered (Figure 1B). Animals were rescued with glucose after the loss of the righting reflex (RR), which precedes the coma state (Haces et al., 2010) by an intraperitoneal (i.p.) bolus of 0.3 ml of 25% glucose in Krebs–Henseleit buffer. Immediately after, an intravenous (i.v.) infusion (25% glucose at 1.5 ml/h during 3 h) was administered through the tail vein using a perfusion pump (Harvard Apparatus 22, South Natick, MA, USA). None of the animals from this group showed EEG isoelectricity (indicative of the hypoglycemic coma). From these animals, one subgroup was euthanized at 6 h after GI (9 h after an insulin injection) and a second subgroup at 24 h after GI (27 h after an insulin injection; Figure 1B). Brains were extracted and prepared for western blotting.

### Induction of Hypoglycemic Coma With Glucose Infusion (Coma)

Animals subjected to coma (Coma), were equally treated as those from the SH + GI group, but after the loss of the RR, hypoglycemia was left to progress to the state of coma (EEG isoelectricity). After 5 min of isoelectricity animals received an i.p. bolus of 0.3 ml of 25% glucose as described for the SH + GI group, and 12 min afterward an intravenous (i.v.) infusion (25% glucose at 1.5 ml/h during 3 h) was administered through the tail vein as described above. The coma period ranged from 6 to 12 min (mean = 8.9 ± 0.34). A minimum period of 6 min coma was chosen because according to our previous studies, shorter coma periods produce very limited neuronal death or none. Therefore, animals showing coma periods of 5 min or less were discarded. Conversely, a maximum period of 12 min coma was selected because respiratory failure can be observed in animals showing longer coma periods. From these animals, one subgroup was euthanized at 6 h and a second subgroup at 24 h after GI (Figure 1C). Brains were extracted and prepared for western blot analysis (6 and 24 h) and histology (24 h). Animals showing seizures were also discarded.

Control animals were treated in parallel with the rest of the experimental groups. They were partially fasted overnight and during the duration of the experimental period and received vehicle solution (0.1% acetic acid) instead of insulin. Glucose was measured at different times after vehicle injection.

### Treatment With D-BHB (Coma + BHB)

Animals exposed to coma were either treated (Coma + BHB) or non-treated (Coma) with 250 or 500 mg/kg total dose of D-BHB (Cat. 298360, Sigma–Aldrich, St. Louis, MO, USA) divided into two intraperitoneal (i.p.) administrations. The first administration of D-BHB (125 or 250 mg/kg) was given 10 min after glucose i.v. the infusion was started and the second at the end of the glucose infusion (3 h after the coma). Animals were euthanized at 6 or 24 h GI as indicated in Figure 1D. Brains were extracted and prepared for western blotting (6 and 24 h) and histology (24 h).

### Determination of D-BHB in Blood

**Figure 2:**
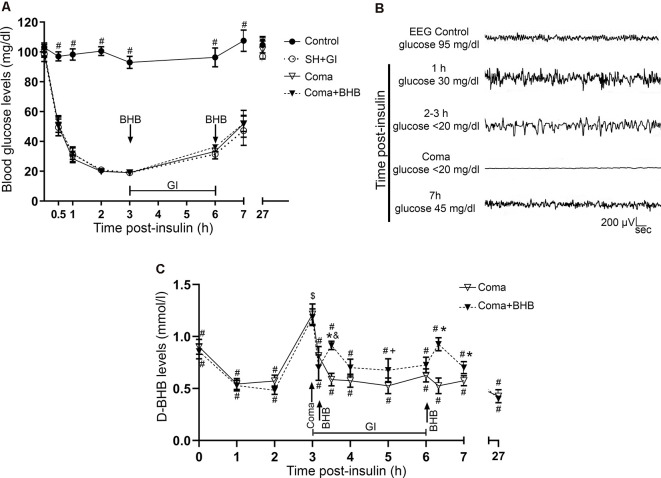
Glucose and D-BHB concentration in blood and EEG recording. **(A)** Blood glucose concentration in control and hypoglycemic animals rescued with glucose (SH + GI), and in animals subjected to coma treated (Coma + BHB) and non-treated (Coma) with D-BHB. Data represent mean ± SEM and were analyzed by one-way ANOVA followed by a Fisher’s *post hoc* test for multiple comparisons. ^#^*p* ≤ 0.05 relative to the hypoglycemia groups, *n* = 6–10. **(B)** Representative EEG recording showing the changes in brain electrical activity in one representative insulin-injected animal before insulin administration (control), during SH (1–3 h after insulin), during the coma, and after GI. **(C)** D-BHB blood levels were determined at different times after insulin injection and GI in rats subjected to coma treated and non-treated with D-BHB. Data represent mean ± SEM and were analyzed by one-way or two-way ANOVA followed by a Fisher’s *post hoc* test for multiple comparisons, for intragroup and intergroup comparisons, respectively. *p ≤ 0.05 vs. the Coma group, ^$^*p* ≤ 0.05 vs. the corresponding 2 h post-insulin value, ^#^*p* ≤ 0.05 vs. the corresponding 3 h value (coma state), ^&^*p* ≤ 0.05 vs. the corresponding 3.1 h value (before D-BHB administration), ^+^*p* ≤ 0.05 vs. the corresponding 3.5 h value (20 min post D-BHB administration), *n* = 7 for each experimental group.

Blood samples were obtained from the tail vein (seven animals per group) and D-BHB was measured using blood glucose and ketone monitoring system (FreeStyle Optium Neo, Abbott Diabetes Care, Limited, Witney, Oxon, UK) and keto strips (FreeStyle Optium β-ketone). Samples were obtained from intact control and fasted animals at different times throughout the experimental period. D-BHB was also determined in blood samples from animals of the Coma and the Coma + BHB groups, before (time 0) and at different times after insulin injection (1 and 2 h), at the time the animals reached the coma, at different times after recovery with glucose or glucose + D-BHB (250 mg/kg), and after the second administration of D-BHB (250 mg/kg), as indicated in Figure 2C. Animals from these groups were identically treated during the hypoglycemia period before recovery and were randomly assigned to each one of the treatments.

### SDS–PAGE and Western Blots

The hippocampus and parietal cortex were dissected and homogenized in 1:10 weight/volume lysis buffer containing: Tris-HCl 50 mM, NaCl 150 mM, SDS 1%, Triton X-100 1%, Sodium deoxycholate 0.5%, PMSF 1 mM, NaPPi 5 mM, Na_3_VO_4_ 2 mM and Complete protease inhibitor cocktail (Roche complete, 1162600, USA), pH 7.5. Proteins were determined by the Lowry method and samples were denaturized in Laemmli buffer. Thirty to forty micrograms of protein was resolved in 10–16% SDS–PAGE and then electroblotted to PVDF membranes. Membranes were blocked in TBS/milk 5% for 1 h and incubated overnight at 4°C with specific primary antibodies: LC3 (1:5,000, Cat. PD014, MBL International Woburn, USA); ATG5 (1:1,000, Cat. S6133158, Santa Cruz Biotechnology, Dallas, TX, USA), p-ULK1 S317 (1:500, MB59600629, My BioSource, San Diego, CA, USA); BECN1 (1:2,000, Cat. 3738), ATG7 (1:1,000, Cat. 2631S), ULK1 (1:6,000, Cat. 8054), p-ULK1 S757 (1:6,000, Cat. 14202), mTOR (1:1,000, Cat. 2983S), p-mTOR S2448 (1:1,000, Cat. 2971), AMPK (1:1,000, Cat. 2532), SQSTM1/p62 (1:2,500, Cat. 5114), were from Cell Signaling Technology (Danvers, MA, USA); Actin (1:7,000, Cat. MAB1501 Chemicon Merck Millipore, Darmstadt, Germany) was used as loading control. The reactions of primary antibodies were detected using the respective horseradish peroxidase, goat anti-rabbit, or goat anti-mouse secondary antibody (Cat. 115035-003 and 115035-062 respectively, Jackson Immunoresearch Laboratories, West Grove, PA, USA). Immunoreactivity was detected by chemiluminescent HRP substrate (Luminata™ Forte, Cat. WBLUF0100, Merck Millipore), using a C-Digit Blot Scanner (LI-COR Biosciences, UK). The optical density of the bands of interest was measured using the ImageJ program. Data were calculated as the protein/actin ratio.

### Histology

Twenty-four hours after the treatments, animals from each group (*n* = 4–7) were anesthetized with an overdose of pentobarbital and intracardially perfused with 0.9% saline solution followed by 4% paraformaldehyde in 0.1 mM phosphate buffer; brains were extracted and transferred to a 20–30% sucrose gradient (24 and 72 h, respectively). Coronal brain sections of 20 and 40 μm were obtained in a cryostat (LEICA CM1510S) for histological analysis.

### Fluoro-Jade B Staining

Slides were covered for 5 and 2 min with 80 and 70% ethanol respectively, they were washed and covered with 0.06% potassium permanganate for 10 min. Sections were incubated for 20 min with 0.0004% FJB (Cat. AB310, Chemicon), dried at 50°C, rinsed with xylol, and covered with permount (Julio-Amilpas et al., 2015). They were observed under an epifluorescence microscope Nikon Eclipse Ci (using AT-EGFP/F filter) and FJB-positive cells were counted in both hemispheres. In the parietal cortex total FJB-positive cells were counted bilaterally in 15 sections separated by 200 μm. In the case of the hippocampus, six sections were used and cells were counted in a 200 μm^2^ area of the crest and the inferior blade of the dentate gyrus using the ImageJ program. Data are reported as the total number of positive cells in both subregions. Cell damage was confirmed by the presence of pyknotic cells after Nissl staining of adjacent sections.

### Immunohistochemistry

Brain sections were permeabilized by 30 min (PBS/Triton-X100 0.9%), washed for 10 min in PBS, and incubated with citrates buffer 0.1% at 58°C for 20 min. They were incubated in PBS/glycine 0.1% for 15 min and then blocked 1 h in PBS/BSA 5%/goat serum 2%/Tween 0.5%/Triton 0.9% at room temperature. Afterward, sections were incubated in primary antibodies against LC3 (1:300) or SQSTM1/p62 (1:200, Cat. ab56416, Abcam, Cambridge, UK) in PBS/BSA 1%/Triton X-100 0.3%/Tween-20 0.05%, for 48 h at 4°C. The slides were washed in PBS and incubated for 2 h with secondary antibody Alexa 488 anti-rabbit and Dry-Light 488 anti-mouse (1:300 Cat. 111-545-144 and 115-485-166 respectively, Jackson Immunoresearch Laboratories, West Grove, PA, USA). Subsequently, cell nuclei were stained with Hoechst 0.001% (Cat. 33258, Sigma–Aldrich). Slides were incubated in Sudan Black B (Cat. 199664, Sigma–Aldrich) for 3 min to decrease background fluorescence. Images were obtained using a confocal microscope ZEISS LSM800 for LC3, SQSTM1/p62, and Hoechst. Images were acquired and processed using the ZEN 3.1 program from Zeiss. Confocal stacks composed from 28 to 35 slices (0.3 μm) were acquired and the maximum projection was obtained from each image (*x*-*y*, *x*-*z*, and *y*-*z* orientations) from three independent experiments.

### Statistical Analysis

All data are expressed as mean ± SEM and were analyzed by the Student’s *t*-test when a comparison between two groups was made, or one-way ANOVA followed by Fisher’s LSD test for multiple comparisons when more than two groups were compared. The time-course in D-BHB blood levels in the Coma and the Coma + BHB groups was compared by two-way ANOVA followed by a Fisher’s LSD multiple comparison test, and the intragroup comparisons were made by one-way ANOVA followed by Fisher’s LSD test for multiple comparisons.

## Results

### Glucose Concentration and Electroencephalogram Recording

Blood glucose levels were measured and electrical brain activity was recorded at different times after insulin injection and GI. Results show a mean basal blood glucose concentration close to 100 mg/dl in all groups. In control animals, glucose concentration was constant during the experimental period (Figure 2A). In hypoglycemic animals (SH + GI, Coma, and Coma + BHB) glucose concentration declined close to 30 mg/dl after 1 h insulin administration and decreased further to 20 mg/dl during the next 2 h before GI. At the end of the GI, glucose levels reached 30 mg/dl and 1 h later they raised to 55 mg/ml; control values were recovered at 24 h. No significant differences were found in blood glucose concentration between the experimental groups (Figure 2A). Figure 2B shows a representative EEG recording obtained before, during, and after the hypoglycemic coma. After 2–3 h insulin administration, electrical brain activity declined to show the high amplitude and low-frequency waves as previously reported (Julio-Amilpas et al., 2015). At this time glucose concentration declined below 20 mg/dl; animals were drowsy and lost their RR. In animals exposed to coma, electrical brain activity was completely suppressed and recovered 1 h after GI. Animals rescued with glucose plus D-BHB (either 250 or 500 mg/kg) showed similar changes in brain electrical activity (not shown).

### Autophagy Dynamics During Hypoglycemia and Glucose Infusion in Animals Subjected to Severe Hypoglycemia or the Hypoglycemic Coma

**Figure 3:**
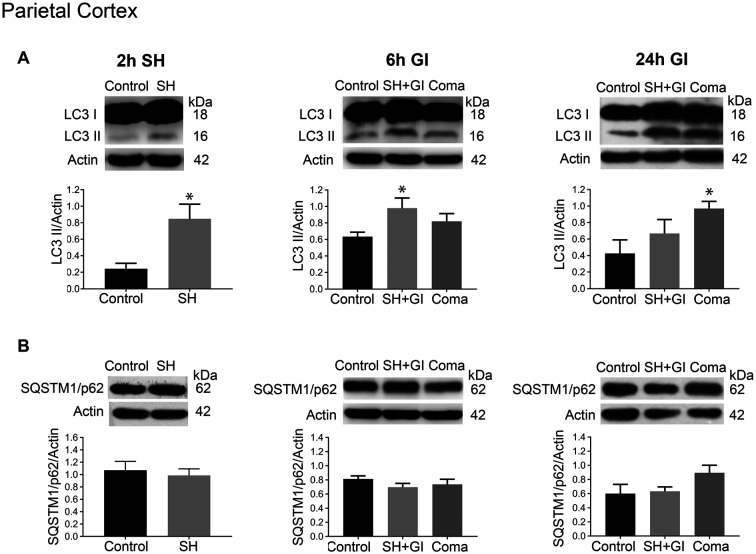
Autophagy dynamics in the parietal cortex induced by severe hypoglycemia and the hypoglycemic coma at different times. Changes in **(A)** LC3-II/Actin and **(B)** SQSTM1/p62/Actin 2 h after SH (2 h SH) and 6 and 24 h after glucose infusion (6 h GI and 24 h GI). Bars represent mean ± SEM. Data were analyzed by one-way ANOVA followed by a Fisher’s *post hoc* test for multiple comparisons. For the 2 h group, a Student’s *t*-test was used for statistical analysis. *p ≤ 0.05 vs. control, *n* = 4–6 for the 2 and 24 h groups and *n* = 3 for the 6 h groups.

The changes in the content of LC3-II and SQSTM1/p62 were determined 2 h after the induction of hypoglycemia and 6 and 24 h after GI in rats exposed to SH + GI or the hypoglycemic coma. In the parietal cortex 2 h after SH, the transformation of LC3-I to LC3-II notably increased, while no change in SQSTM1/p62 was observed. At 6 h after GI, LC3-II remained significantly elevated and returned to control levels at 24 h in the SH + GI group (Figure 3A). As the increase in LC3-II was not accompanied by a decrease in SQSTM1/p62 (Figure 3B), results suggest that the autophagic flux is blocked and that increased LC3-II results from autophagosome accumulation. In animals subjected to coma, LC3-II showed a moderate non-significant increase at 6 h after GI, but it increased notably at 24 h. As in the case of SH + GI, augmented LC3-II was not accompanied by a decline in SQSTM1/p62, suggesting autophagic flux impairment at 24 h after GI (Figures 3A,B). In agreement with these observations, LC3 immunoreactivity increased 24 h after GI in the Coma group (Figure 5C).

**Figure 4:**
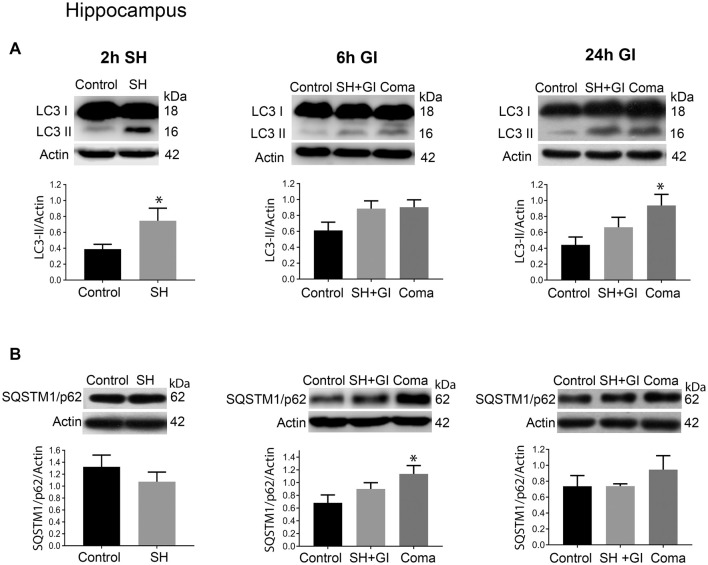
Autophagy dynamics in the hippocampus induced by severe hypoglycemia and the hypoglycemic coma at different times. Changes in **(A)** LC3-II/Actin and **(B)** SQSTM1/p62/Actin 2 h after SH (2 h SH) and 6 and 24 h after glucose infusion (6 h GI and 24 h GI). Bars represent mean ± SEM. Data were analyzed by one-way ANOVA followed by a Fisher’s *post hoc* test for multiple comparisons. For the 2 h group, a Student’s *t*-test was used for statistical analysis. *p ≤ 0.05 vs. control, *n* = 4–6 for the 2 and 24 h groups and *n* = 3 for the 6 h groups.

**Figure 5:**
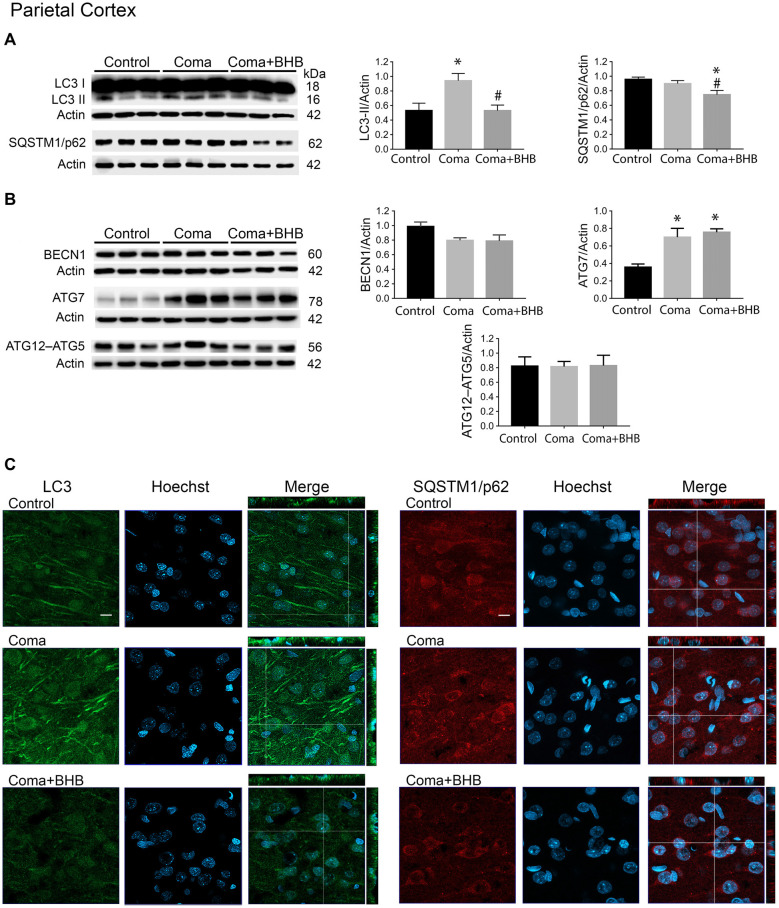
Effect of D-BHB on autophagy-related proteins in the parietal cortex 24 h after the hypoglycemic coma. Changes in **(A)** LC3-II/Actin and SQSTM1/p62/Actin, **(B)** BECN1/Actin, ATG7/Actin, and ATG12–ATG5/Actin. **(C)** Confocal representative images of immunoreactivity to LC3 and SQSTM1/p62 in animals treated and non-treated with D-BHB (500 mg/kg). Orthogonal images (x-z upper) and y-z (right) orientations are shown. Data are expressed as mean ± SEM. Statistical analysis was performed by one-way ANOVA followed by the Fisher *post hoc* test for multiple comparisons. *p ≤ 0.05 vs. control, ^#^*p* < 0.05 vs. coma, *n* = 4–5 control, *n* = 5–6 Coma, *n* = 5 Coma + BHB. Scale bar = 10 μm.

**Figure 6:**
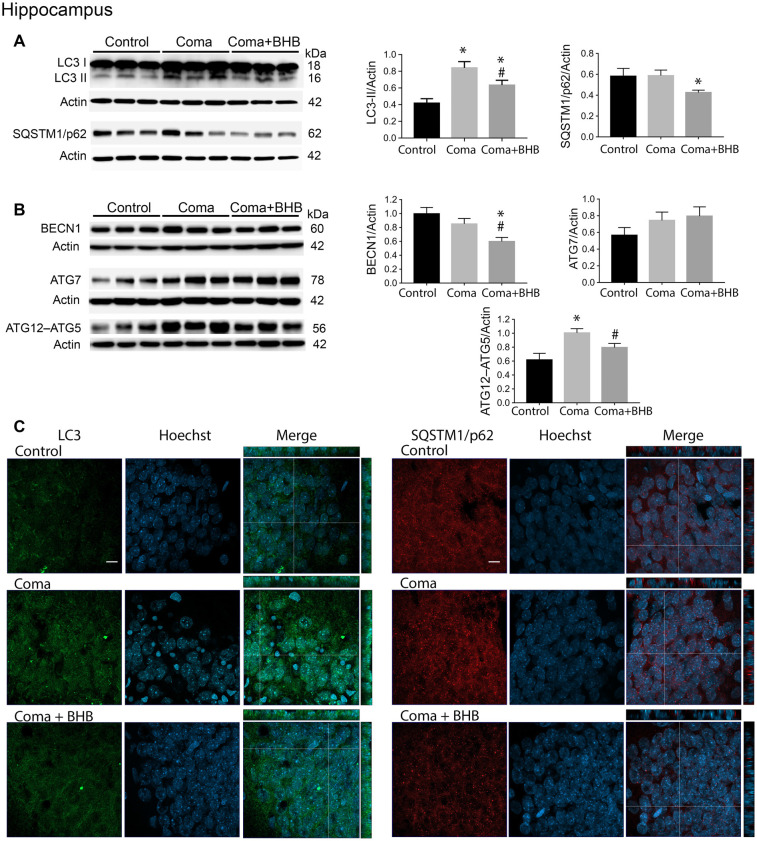
Effect of D-BHB on autophagy-related proteins in the hippocampus 24 h after the hypoglycemic coma. **(A)** Changes in LC3-II/Actin and SQSTM1/p62/Actin, **(B)** BECN1/Actin, ATG7/Actin, and ATG12–ATG5. **(C)** Confocal representative images of immunoreactivity to LC3 and SQSTM1/p62 in animals treated and non-treated with D-BHB (500 mg/kg). Orthogonal images (x-z upper) and y-z (right) orientations are shown. Data are expressed as mean ± SEM. Statistical analysis was performed by one-way ANOVA followed by the Fisher *post hoc* test for multiple comparisons. *p ≤ 0.05 relative to control, ^#^*p* < 0.05 relative to coma, *n* = 4–5 control, *n* = 5–6 Coma, *n* = 5 Coma + BHB. Scale bar = 10 μm.

Similar to the parietal cortex in the hippocampus LC3-II significantly increased 2 h after hypoglycemia, while no significant change in SQSTM1/p62 was found (Figures 4A,B). In rats subjected to SH + GI, no significant change in LC3-II or SQSTM1/p62 was observed at 6 h and 24 h (Figure 4B). In contrast, in the Coma group, a significant increase in LC3-II was found at 24 h after GI, while SQSTM1/p62 content showed no reduction suggesting deficient autophagic degradation. In agreement with these observations, augmented immunoreactivity against LC3 was observed in brain sections from animals exposed to coma, in the inferior blade (not shown) and the crest of the dentate gyrus at 24 h after GI (Figure 6C).

Altogether, these data suggest that autophagosomes accumulate during the hypoglycemic period due to deficient autophagic degradation. After GI the autophagic flux is restored in the SH + GI group, while in rats experiencing a period of coma autophagosomes accumulate at 24 h, likely due to impaired autophagic flux. No changes in the LC3-I band were observed at 2 h or 6 h in the SH + GI and Coma groups (data not shown), while at 24 h there was a slight significant increase in the LC3-I band in the hippocampus of rats experiencing coma but not in the cortex (Supplementary Figure S2).

### D-BHB Administration After the Hypoglycemic Coma Stimulates the Autophagic Flux and Increases Cell Survival

In a previous study, we have reported that D-BHB stimulates the autophagic flux and prevents neuronal death in cortical cultured neurons exposed to glucose deprivation and glucose reintroduction (Camberos-Luna et al., 2016). Hence, we aimed to test whether the KB exerts the same effect in the hypoglycemia model. Rats exposed to the hypoglycemic coma were recovered with glucose and D-BHB 250 mg/kg (two doses of 125 mg/kg) and were analyzed at 6 and 24 h. D-BHB treatment did not affect 6 h on LC3-II or SQSTM1/p62 in the cortex or the hippocampus (data not shown). However, at 24 h D-BHB reduced the increase in LC3-II induced by coma in both brain regions, while no change in SQSTM1/p62 was found (Supplementary Figures S1A,B). These results are consistent with immunohistochemistry data, showing reduced LC3 immunoreactivity in rats treated with D-BHB (Supplementary Figures S1A,B). When 500 mg/kg D-BHB (two administrations of 250 mg/kg each) was administered, the transformation of LC3-I to LC3-II significantly diminished relative to animals exposed to coma in both the cortex and the hippocampus (Figures 5A, 6A). Also, a significant reduction of SQSTM1/p62 was observed in D-BHB-treated rats (Figures 5B, 6B), suggesting that at higher doses D-BHB stimulates the autophagic flux in both regions. In agreement with these results, immunohistochemistry analysis showed reduced immunoreactivity to LC3 and SQSTM1/p62 in the parietal cortex and the dentate gyrus of animals treated with 500 mg/kg of D-BHB (Figures 5C, 6C). Confocal *Z*-stacks images show that LC3 and SQSTM1/p62 are present in the cytoplasm surrounding the nucleus and also in neurites. In animals subjected to coma LC3 and SQSTM1/p62 immunoreactivity augment, while in D-BHB-treated animals, immunofluorescence is less intense and more diffuse. Orthogonal images from the *x*-*z* and *y*-*z* orientations confirm that LC3 and SQSTM1/p62 are located in the cytoplasm.

D-BHB treatment did not affect LC3-I in the cortex relative to the control and Coma groups and did not reduce the increase in LC3-I induced by the coma in the hippocampus (Supplementary Figure S2).

**Figure 7:**
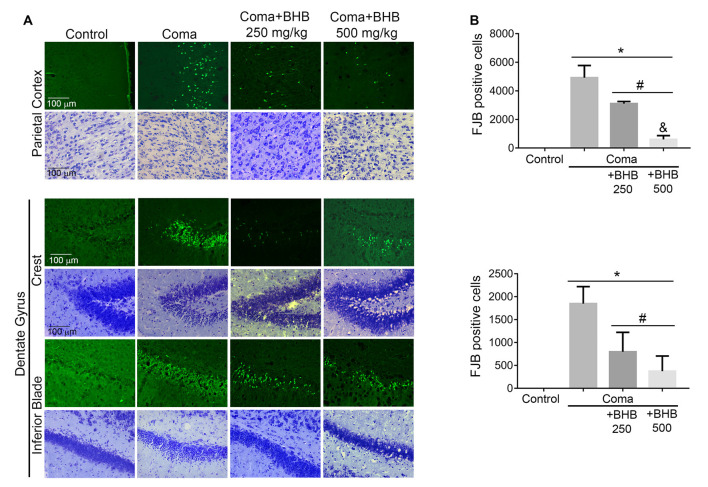
Effect of D-BHB treatment on cell survival in the cortex and the hippocampus of rats exposed to the hypoglycemic coma. **(A)** Representative micrographs of FJB-positive cells and cresyl violet-stained sections of the parietal cortex and dentate gyrus. **(B)** The number of FJB-positive cells in control and hypoglycemic animals treated and non-treated with 250 or 500 mg/kg D-BHB in the parietal cortex and the dentate gyrus of the hippocampus. Data are expressed as mean ± SEM and were analyzed by one-way ANOVA followed by Fisher *post hoc* test for multiple comparisons. *p ≤ 0.05 vs. control, ^#^*p* ≤ 0.05 vs. coma, ^&^*p* ≤ 0.05 vs. 250 mg/kg, *n* = 6 control, *n* = 7 coma, *n* = 4 Coma + BHB.

Then we tested the effect of D-BHB administration on cell survival. In previous studies, we have reported that a short period of coma induces neuronal death in the cerebral cortex and the dentate gyrus (Languren et al., 2019). FJB and cresyl violet staining were used to evidence neuronal damage. At 24 h after glucose infusion, degenerating cells labeled with FJB were observed in the parietal cortex, mainly in the superficial and medium layers (II–IV), and in the hippocampus, primarily in the crest and the inferior blade of the dentate gyrus (Figure 7A). Adjacent cresyl violet-stained sections showed dark shrunk cells with pyknotic nuclei in the same location where FJB-positive cells were present (Figure 7A).

D-BHB treatment reduced the number of FJB-positive cells in both cerebral regions (Figure 7B). In the parietal cortex, protection by D-BHB was dose-dependent. A 36% reduction in the number of FJB-labelled cells was observed when animals were treated with two doses of 250 mg/kg D-BHB after the hypoglycemic coma. When 500 mg/kg D-BHB was used, the number of FJB-positive cells was reduced by 87% (Figure 7B). Accordingly, cresyl violet-stained sections of animals treated with D-BHB showed cells with a light cytoplasm and morphologically similar to those in control animals. In the hippocampus, the number of degenerating cells was reduced by 57 and 80%, when animals were treated with 250 or 500 mg/kg of D-BHB, respectively, although no significant difference was found between the two doses (Figure 7B). These results demonstrate that D-BHB can reduce neurodegeneration when administered post the hypoglycemic coma and that a 500 mg/kg total dose is more effective.

D-BHB blood levels were determined at different times in intact control animals, fasted animals, and animals subjected to coma treated and non-treated with D-BHB. As expected, fasting-induced a significant increase in D-BHB blood levels to 0.9 ± 0.075 mM relative to intact controls (0.6 ± 0.057), and these levels were maintained throughout the whole experimental period, recovering control values 27 h later (0.56 ± 0.13; not shown). One-hour after insulin administration, when glucose blood levels decreased close to 30 mg/dl, D-BHB decayed to 0.5 mM (Figure 2C). When glucose further decreased below 20 mg/dl and animals reached the coma state, D-BHB blood concentration significantly increased up to 1.2 mM but declined soon after the coma to 0.8–0.7 mM (Figure 2C). To this stage, both animal groups showed a similar behavior suggesting they similarly reacted to insulin. During recovery, 30 min after animals were rescued with glucose alone, D-BHB decreased further (0.58 ± 0.059) and its levels remained low and statistically different from the coma value during the whole glucose infusion period. In contrast, in animals rescued with glucose and D-BHB (250 mg/kg), KB levels did not decline but significantly increased to 0.91 ± 0.040 after 20 min, and progressively declined to 0.7 mM during the glucose infusion period (Figure 2C). Twenty minutes after the second administration of D-BHB its blood levels increased again but rapidly declined after 30 min. Twenty-four hours after glucose infusion, D-BHB levels declined close to control values in both groups (Figure 2C). Altogether, these results suggest that D-BHB endogenous production is stimulated during the coma state, but is rapidly consumed during GI. Under these conditions, the exogenous administration D-BHB during the recovery period significantly increases its blood levels relative to animals rescued with glucose alone.

### Autophagy Initiation After the Hypoglycemic Coma

To investigate whether the elevation of LC3-II induced by the hypoglycemic coma is associated with autophagy induction, the changes in autophagy initiation proteins were determined. In the parietal cortex, no significant changes in BECN1 were observed. The antibody used against ATG5, detects a 56 kDa band corresponding to the ATG12–ATG5 conjugate and a 32 kDa band corresponding to ATG5 alone. In the present conditions, the 32 kDa band showed a very low intensity and is not shown. No changes were detected in the ATG12–ATG5 conjugate relative to control rats, suggesting no activation of autophagy at this time (Figure 5B). However, a significant increase in ATG7 was found. In the hippocampus no changes in BECN1 were observed, ATG7 showed a trend to increase and ATG12–ATG5 conjugate was significantly elevated, suggesting increased autophagosome formation (Figure 6B).

Then we tested the effect of D-BHB on autophagy initiation proteins and observed no change in BECN1, ATG7, and ATG12–ATG5 conjugate in the parietal cortex relative to the Coma group (Figure 5B), while in the hippocampus D-BHB treatment induced no change in ATG7 content, but produced a significant decrease in BECN1 and ATG12–ATG5 conjugate compared to the Coma group (Figure 6B). These results suggest that D-BHB inhibits autophagy initiation in the hippocampus.

### Role of mTOR and AMPK Activation on Autophagy Initiation After the Hypoglycemic Coma

**Figure 8:**
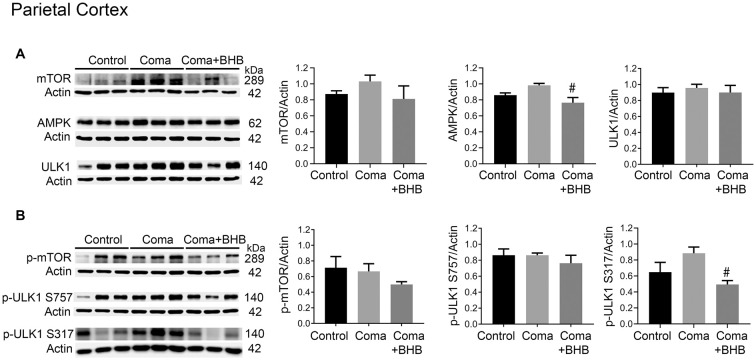
mTOR and AMPK activity in the parietal cortex of animals treated and non-treated with D-BHB 24 h after the hypoglycemic coma. **(A)** Total levels of mTOR, AMPK, and ULK1. **(B)** Phosphorylation levels of mTOR S2448, ULK1 S757, and ULK1 S317. Data are expressed as mean ± SEM. Statistical analysis was performed by one-way ANOVA followed by the Fisher *post hoc* test for multiple comparisons. ^#^*p* ≤ 0.05 vs. coma. *n* = 4–5 control, *n* = 5–6 coma, *n* = 4–5 Coma + BHB.

**Figure 9:**
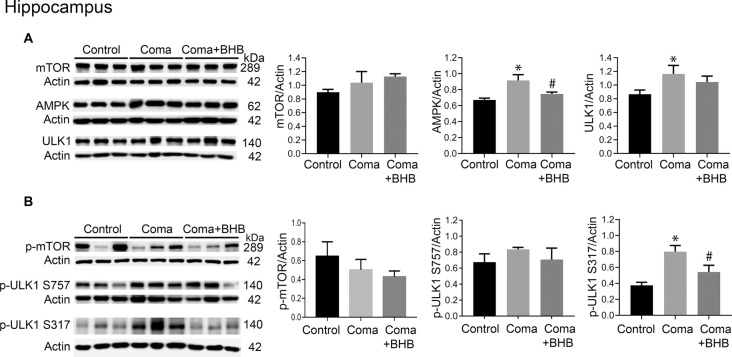
mTOR and AMPK activity in the parietal cortex of animals treated and non-treated with D-BHB 24 h after the hypoglycemic coma. **(A)** Total levels of mTOR, AMPK, and ULK1. **(B)** Phosphorylation levels of mTOR S2448 and ULK1 S757 and ULK1 S317. Data are expressed as mean ± SEM. Statistical analysis was performed by one-way ANOVA followed by the Fisher *post hoc* test for multiple comparisons. *p ≤ 0.05 vs. control, ^#^*p* ≤ 0.05 vs. to coma. *n* = 4–5 control, *n* = 5–6 coma, *n* = 4–5 Coma + BHB.

Activation of AMPK and inhibition of the mTOR complex leads to autophagy initiation. Therefore, the levels of phosphorylation of the downstream target of these two kinases, ULK1 were determined 24 h after the coma as an index as of their activity. No significant changes in total mTOR, p-mTOR S2448, total ULK1, and p-ULK1 S757 were found in the cortex (Figures 8A,B), suggesting that mTOR activity is not inhibited at this time. Total AMPK showed no change and p-ULK1 S317 tended to increase (Figures 8A,B). In the hippocampus no changes in total mTOR, p-mTOR S2448 and p-ULK S757 were present, but a significant increase in total ULK1 was found (Figures 9A,B). In contrast, total AMPK and p-ULK1 S317 significantly increased after the coma (Figures 9A,B). Overall, these results agree with those of ATG proteins and suggest that at 24 h autophagy is initiated mainly in the hippocampus in an AMPK activity-dependent manner.

### Effect of D-BHB on mTOR and AMPK Activation After the Hypoglycemic Coma

The effect of 500 mg/kg of D-BHB was tested in mTOR and ULK1 phosphorylation. No changes in p-mTOR S2448 and p-ULK1 S757 were found neither in the cortex nor the hippocampus of D-BHB-treated animals as compared to the Coma group (Figures 8B, 9B). D-BHB treatment decreased total AMPK and prevented the increase in p-ULK1 S317 induced by the hypoglycemic coma in both brain regions, suggesting that in D-BHB-treated rats AMPK is less activated (Figures 8A,B, 9A,B). Altogether, these results suggest that D-BHB treatment attenuates autophagy activation.

## Discussion

Under nutrient-limiting conditions, cells activate autophagy to restore cell homeostasis (Mizushima et al., 2010). However, during conditions of severe energy failure such as ischemia, hypoxia, and cerebral trauma, neuronal death can result from dysfunctional (Carloni et al., 2008; Sarkar et al., 2014) or excessive autophagy (Shi et al., 2012). In contrast, it has been reported that during mild hypoxia and ischemic preconditioning, autophagy is activated to protect cells by removing damaged organelles and proteins (Sheng et al., 2012; Sun et al., 2018). We have previously reported that glucose depletion in cortical cultured neurons activates autophagy, but after glucose reintroduction deficient autophagic degradation is triggered due to calpain-mediated lysosomal dysfunction contributing to neuronal death (Geronimo-Olvera et al., 2017).

Evidence about autophagy dynamics in an *in vivo* model of severe hypoglycemia is still lacking. We have addressed this question and the present findings suggest for the first time, that autophagosomes accumulate during a period of SH relative to control animals, likely due to deficient autophagic degradation in the parietal cortex and the hippocampus. When glucose was infused after SH a significant increase in autophagosome formation relative to controls animals was observed at 6 h, but it was statistically significant only in the parietal cortex. This increase was transient and returned to control values at 24 h, suggesting that basal levels of autophagy were restored at this time. In contrast, animals experiencing a period of coma showed a non-significant elevation in LC3-II at 6 h relative to control values, but at 24 h LC3-II further and significantly increased. The increase in LC3-II was not different from the SH group but it was different to control rats in both brain regions, suggesting that autophagy does not recover basal values when rats experience a period of coma. As no decrease in SQTSM1/p62 was observed, it is suggested that autophagosome accumulation results from deficient autophagic flux. On the other hand, it was observed that LC3-I abundance significantly increased in the hippocampus but not in the cortex, suggesting the up-regulation of this protein after GI or its deficient degradation, possibly due to impaired proteasomal activity (Gao et al., 2010). Further experiments are needed to identify the mechanism involved.

The differential autophagy dynamics observed after SH and the hypoglycemic coma might be related to the duration and the intensity of energy failure. Early studies revealed that the levels of phosphocreatine and ATP are not altered during non-coma hypoglycemia, while isoelectricity triggers a severe unbalance of the energy state leading to energy depletion (Ferrendelli and Chang, 1973; Lewis et al., 1974). On the other hand, previous studies have shown that GI after non-coma SH increases ROS production and 3-nitrotyrosine protein residues, but neuronal death is limited to a few scattered cells in the cerebral cortex (Haces et al., 2010; Amador-Alvarado et al., 2014; Julio-Amilpas et al., 2015). In contrast, it is well known that GI after the coma induces oxidative stress and severe oxidative damage, which is highly involved in neuronal death in the cortex and the hippocampus (Suh et al., 2003, 2007). Thus, it is feasible that non-coma hypoglycemia transiently activates autophagy to remove damaged organelles and other cellular components to reestablish cell homeostasis and prevent neuronal death, while the excessive accumulation of oxidatively damaged molecules after the coma, will exacerbate autophagosome formation and impair the autophagic flux.

On the other hand, the present results indicate that autophagy is activated in the hippocampus 24 h after GI in an AMPK-dependent mTOR-independent manner in rats exposed to coma. A moderate increase in ATG7 and a significant elevation in the ATG12–ATG5 conjugate relative to the control group were found. As these proteins are essential for autophagosome formation and elongation, respectively, these results suggest that autophagy is activated in the hippocampus 24 after GI. In agreement with these results, a significant increase in p-ULK1 S317 was found relative to control animals suggesting that autophagy is initiated by AMPK activation. In the cortex, autophagy initiation was not so evident. A significant elevation of ATG7 was found but neither in ATG12–ATG5 conjugate nor in p-ULK1 S317, although a trend to augment was observed. The differences observed in autophagy activation in the cortex and the hippocampus, might be related to the high energy demand of the latter, due to its role in synaptic plasticity and its high vulnerability to hypoglycemic injury (Auer et al., 1984).

The mechanisms leading to AMPK activation in the present conditions were not explored and need further investigation. The stimulation of calcium-calmodulin kinase β (CAMKKβ) by increased intracellular calcium might be involved, as calcium homeostasis is lost during severe energy deprivation (Sun et al., 2016). Also, liver kinase B1 (LKB1), an upstream AMP kinase, might have a role as its levels of phosphorylation increase after brain ischemia (Li et al., 2007). Also, AMPK activity can be stimulated by reactive oxygen and nitrogen species, and CAMKKβ and LKB1 can be activated by nitric oxide and peroxynitrite, respectively (Cardaci et al., 2012; Li et al., 2013; Xia et al., 2018).

It is well known that during glucose limiting conditions other energy substrates can be used by the brain, such as the KB. Increasing evidence supports the protective effect of KB exogenous intake or ketosis induction against acute brain injury (Camberos-Luna and Massieu, 2020). We have previously shown that D-BHB administration during hypoglycemia prevents ROS production in the cortex and the hippocampus and diminishes neuronal death (Julio-Amilpas et al., 2015). Here, we investigated whether the administration of D-BHB post-hypoglycemic coma reduces neuronal damage and improves autophagic flux, as previously observed in cortical cultured neurons (Camberos-Luna et al., 2016). According to the results, a total dose of 250 mg/kg D-BHB reduced the increase in LC3-II but was unable to stimulate the degradation of SQSTM1/p62. In contrast, 500 mg/kg of D-BHB significantly reduced LC3-II and SQSTM1/p62 in the parietal cortex and the hippocampus, suggesting the improvement of autophagic cargo degradation. These observations correlated with a reduced number of FJB-positive cells in both brain regions, suggesting that the protective effect of D-BHB is associated at least in part, with the re-establishment of the autophagic flux, as previously reported (Camberos-Luna et al., 2016; Montiel et al., 2020). Importantly, results also show that D-BHB treatment attenuates autophagosome formation in the hippocampus at late stages after GI, supporting the idea that dysfunctional and/or excessive autophagy contributes to neuronal death.

The decrease in LC3-II and SQSTM1/p62 induced by D-BHB treatment can also result from diminished autophagy activation. According to the results, D-BHB did not affect BECN1, ATG7, and ATG12–ATG5 conjugate in the parietal cortex, thus it mainly improved autophagic degradation in this cerebral region. However, in the hippocampus, D-BHB prevented the phosphorylation of ULK1 S317 by AMPK suggesting less AMPK activation. This result correlated with diminished BECN1 and ATG12–ATG5 conjugate supporting that D-BHB attenuates the initiation of autophagy in this region. These results agree with recent findings in the rat striatum after NMDA-induced excitotoxicity (Montiel et al., 2020). Decreased activation of AMPK by D-BHB treatment is possibly related to improved mitochondrial metabolism and ATP synthesis and decreased ROS production (Maalouf et al., 2007; Julio-Amilpas et al., 2015; Marosi et al., 2016).

According to the determination of D-BHB blood levels, fasting-induced mild ketosis as it increased D-BHB up to 0.9 mM. However, it rapidly declined after insulin administration suggesting it is consumed by the brain during hypoglycemia. Results also indicate that during severe hypoglycemia, when animals reach the coma, the endogenous KB production is stimulated but declines soon after the coma. Animals from both groups behaved similarly during the hypoglycemia period before glucose recovery suggesting the similarly reacted to insulin. However, it remains to be determined whether a differential response to insulin affects neuronal survival and autophagy. According to the present data, the exogenous administration of D-BHB immediately after the coma, was able to increase its levels close to fasting values. Notably, D-BHB concentration declined 30 min after its first and second administration, suggesting its utilization during the recovery period. These results agree with a previous study showing that D-BHB administration during hypoglycemia before the coma, increases its levels close to fasting values providing the brain with an alternative substrate to glucose (Julio-Amilpas et al., 2015).

The contribution to neuronal survival of the endogenous D-BHB production and utilization, resulting from insulin administration, cannot be disregarded. In the present experimental conditions, it possibly contributes to the limited neuronal death associated with a brief period of coma (Haces et al., 2010; Languren et al., 2019), as compared to longer periods (30–60 min), where neuronal death is extensive and distributed in the cortex and all layers of the hippocampal formation (Auer et al., 1984; Suh et al., 2007). Nonetheless, according to the present data, additional D-BHB exogenous supplementation during GI, associates with improved cell survival, as a lower number of degenerating cells were observed in D-BHB-treated rats, and this effect was dose-dependent.

The protective effect of exogenous KB administration against acute brain injury has been demonstrated in several *in vivo* models (Suzuki et al., 2001, 2002; Haces et al., 2008; Julio-Amilpas et al., 2015; Montiel et al., 2020), and this study adds new knowledge about the role of autophagy as a possible mechanism involved. Protection protocols using ketosis induction by diet approaches as the ketogenic diet or medium-chain triglycerides supplementation, relay on the up-regulation of monocarboxylate transporters to favor KB oxidation by the brain. In contrast, the exogenous administration of KB or KB derivatives rapidly increases their blood concentration but require repetitive or continuous administration to sustain elevated KB blood levels (Camberos-Luna and Massieu, 2020). It remains to be determined whether there is an up-regulation of MCT transporters in the present conditions, which can facilitate KB uptake in the brain, as it has been reported after ischemia and cerebral trauma (Tseng et al., 2003; Prins et al., 2004; Zhang et al., 2005; Prins and Giza, 2006; Prins, 2008; Moreira et al., 2009), and recently after severe hypoglycemia in female rats (Uddin et al., 2020).

In conclusion, the present study reports for the first time, that autophagy follows different dynamics during glucose recovery in animals experiencing SH or hypoglycemic coma, and that it is differentially activated in the cortex and the hippocampus as a response to the initial energy failure and recovery. Also, results support that D-BHB treatment is associated with the stimulation of the autophagic flux in the cortex and the hippocampus and with the attenuation of autophagy in the latter. These effects might be involved in the protective effect exerted by D-BHB against hypoglycemic neuronal death. These data increase our knowledge about the adaptive brain responses to severe hypoglycemia, and the role of D-BHB as alternative energy fuel to glucose in the modulation of these responses.

## Data Availability Statement

The raw data supporting the conclusions of this article will be made available by the authors, without undue reservation.

## Ethics Statement

The animal study was reviewed and approved by Comité Interno para el Cuidado y Uso de Animales de Laboratorio (CICUAL) from Instituto de Fisiología Celular (protocol number IFC LMT16020).

## Author Contributions

CT-E, TM, and MF-M performed all the experiments. CT-E performed all western blot, histology, and immunohistochemistry analysis. CT-E and LM analyzed all data and wrote the article. CT-E, TM, and LM conceived the study, designed the experiments, and participated in all discussions.

## Conflict of Interest

The authors declare that the research was conducted in the absence of any commercial or financial relationships that could be construed as a potential conflict of interest.
