# Supplementary figures and images for: Effect of β-Hydroxybutyrate on Autophagy Dynamics During Severe Hypoglycemia and the Hypoglycemic Coma
Source: Front Cell Neurosci. 2020 Sep 23;14:547215. doi: 10.3389/fncel.2020.547215 (PMC7538649; doi:10.3389/fncel.2020.547215)

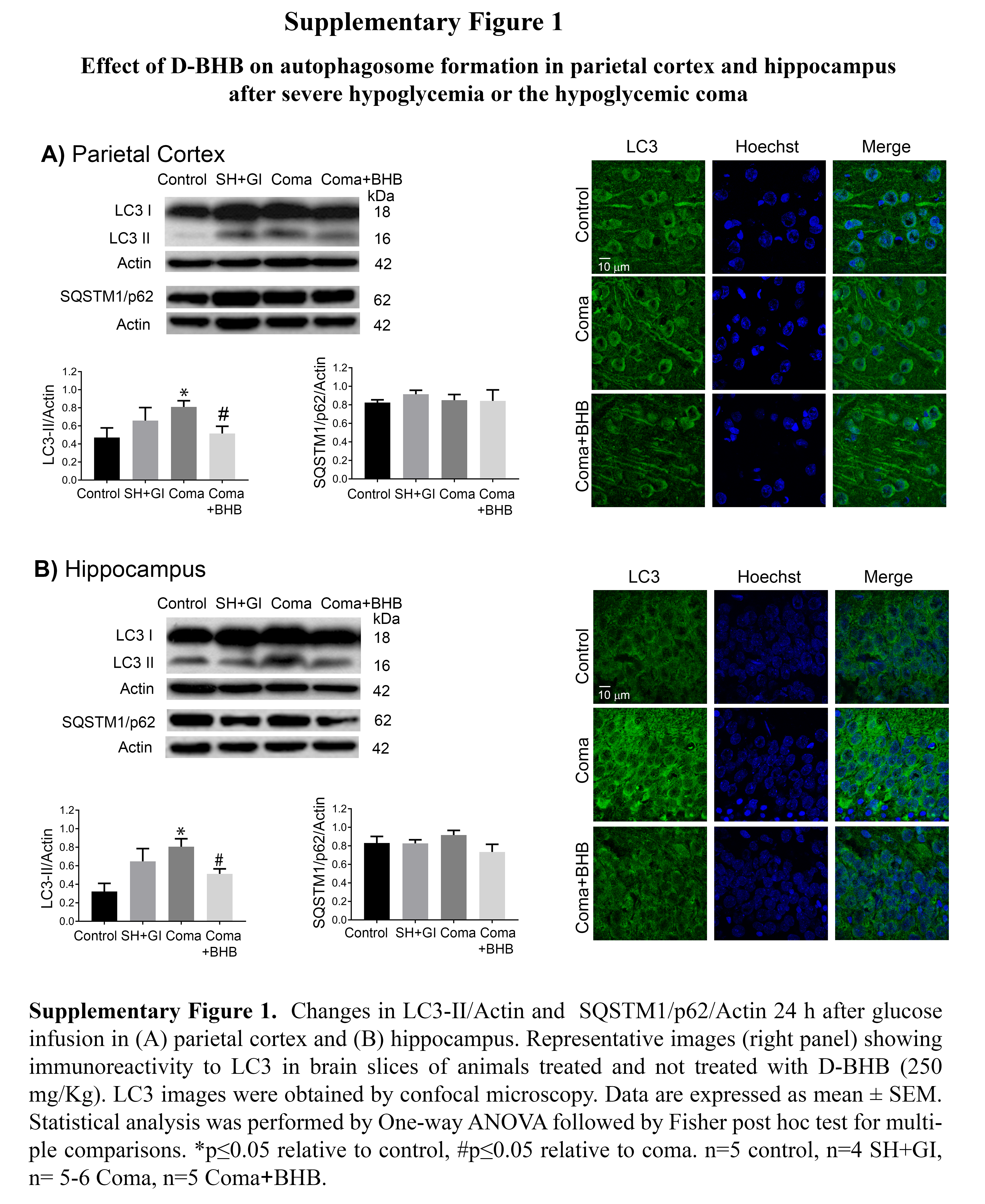

Supplement: Supplementary file 1 [file Image_1.TIF]

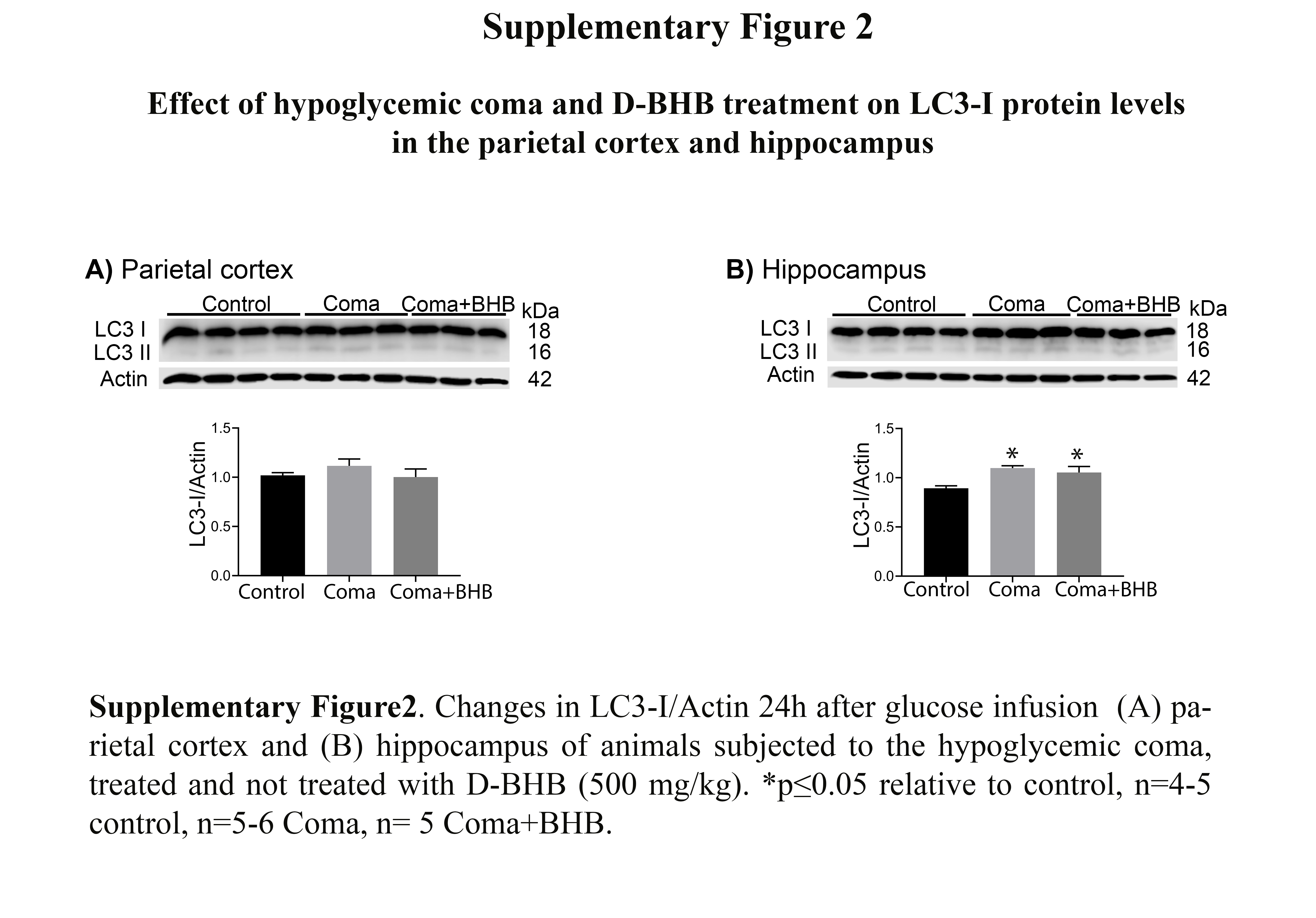

Supplement: Supplementary file 2 [file Image_2.TIF]
